# Quantum error-correction using humming sparrow optimization based self-adaptive deep cnn noise correction module

**DOI:** 10.1038/s41598-024-65182-2

**Published:** 2024-06-21

**Authors:** Umesh Uttamrao Shinde, Ravikumar Bandaru

**Affiliations:** Department of Mathematics, School of Advanced Sciences, VIT-AP University, Besides AP Secretariate, Amaravati, Andhra Pradesh 522237 India

**Keywords:** Humming sparrow optimization, Self adaptive deep CNN, Quantum error correction, Heavy hexagonal code and quantum computing, Engineering, Mathematics and computing

## Abstract

The error correction model’s main purpose in heavy hexagonal quantum codes is to improve their reliability for quantum computing applications. Existing challenges include finding the optimal decoder for quantum error correction in heavy hexagonal codes. This research propels the frontier of quantum error correction, with a specific focus on tailoring topological quantum error-correcting codes for the unique challenges posed by superconducting qubits in quantum computers. In response, this research harnesses the power of deep learning, presenting a Humming sparrow optimization based self-adaptive deep CNN (HSO-based SADCNN) model designed for heavy hexagonal codes. This decoder incorporates a Self-adaptive Deep CNN (SADCNN) Noise Correction Module, a sophisticated component to refine error correction. The proposed decoder’s efficacy is rigorously evaluated across varying code distances (three, five, and seven) using the Humming Sparrow Optimization (HSO) algorithm. HSO, intricately designed to fine-tune the SADCNN decoder, significantly enhances its error correction capabilities for heavy hexagonal quantum codes. The algorithm seamlessly integrates advantageous characteristics of herding and tracing from Humming Bird optimization and Sparrow search optimization, representing a critical stride in advancing the reliability of quantum computing applications, particularly within the intricate domain of heavy hexagonal quantum codes. Based upon the achievements, the Training Percentage (TP) 90 metrics demonstrate significant progress, boasting a commendable accuracy of $97.35\%$, coupled with reduced logical error probability and a diminished bit error rate, marked at 5.51 and 3.72, respectively.

## Introduction

The historical origin of quantum error-correcting codes traces back to 1995, when Shor marked a groundbreaking milestone by introducing the world’s first quantum ECC. This pioneering achievement was based on the quantum analog of the repetition code^4^. Since that pivotal moment, the field has experienced swift progress, witnessing significant advancements in the theoretical underpinnings of quantum codes. Within the quantum computation and communication domain, Quantum Error-Correcting Codes (QECC) emerge as a fundamental cornerstone, assuming a pivotal role in surmounting the challenges posed by quantum decoherence^6^. Simultaneously, there has been a growing interest in fault detection and diagnosis within the domain of process systems engineering, driven by the imperative to ensure safe and seamless system operation^3^. Despite the conceptual foundation of Quantum Error Correction (QEC) and its role in enabling scalable quantum computation, the pursuit of quantum computational prowess comes with a non-trivial computational resource cost^5,7^. Contemporary quantum devices often exhibit error rates perilously close to, or surpassing, the fault-tolerance threshold, limiting the efficacy of QEC. Quantum Error Mitigation (QEM) has emerged as a promising concept, offering new avenues for more robust quantum computing^8^.

However, constructing quantum codes with favorable parameters remains a formidable challenge, hindering the development of a universally applicable method^6^. Quantum computing^32^, leveraging subatomic particles for information storage and manipulation, operates under the principles of quantum mechanics, introducing quirks that result in an alarming level of unreliability compared to classical counterparts. Quantum error correction emerges as a solution to address these challenges, offering the prospect of unlocking the full potential of quantum computers^9^.

Quantum information theory uses entanglement purification to improve the quality of entangled quantum states, which is crucial to quantum communication and computation^34^. Existing quantum error correction models encounter significant challenges that impede the development of practical and scalable quantum computers. One major hurdle is the substantial resource overhead required, as error correction necessitates redundant qubits, exacerbating the limitations of available quantum resources^10–12^. Decoherence and high fault rates also pose significant threats, diminishing the effectiveness of error correction codes. The act of measurement itself introduces errors, known as measurement-induced errors, further complicating the error correction process. Fault-tolerant thresholds must be maintained, demanding lower error rates for physical qubits, gate operations, and measurements^13,33^. Adapting error correction to diverse quantum hardware architectures, real-time monitoring and correction of errors during dynamic computations and ensuring scalability as quantum computers expand in size are additional formidable challenges^16^. Addressing these issues is crucial for unlocking the true potential of quantum computing in solving complex problems. Researchers are actively exploring novel algorithms and hardware improvements to surmount these challenges and advance the field of quantum error correction^17–20^.

Deep Quantum Error Correction in machine learning involves leveraging deep learning techniques to enhance the process of identifying and correcting errors in quantum computations. Quantum states are represented as inputs to a neural network, which is trained to recognize patterns associated with errors in these states^21–23^. Through the training process, the neural network learns to make predictions about errors in quantum states, enabling it to guide a subsequent error correction process^24^. This approach can potentially improve the efficiency of error correction in quantum computations by harnessing the power of deep learning to handle the intricate and complex nature of quantum systems. The application of deep learning to quantum error correction is an evolving area of research to develop more robust and scalable quantum computing systems^25^.

The main contribution of this research lies in developing HSO-based SADCNN for HHCs in quantum error correction. This decoder incorporates a Self-adaptive Deep CNN Noise to address finding an optimal decoder for quantum error correction in superconducting qubits. The research systematically evaluates the proposed decoder’s effectiveness across different code distances and introduces a significant advancement by combining the strengths of Humming Bird and Sparrow search optimization methods. The ultimate goal is to enhance error correction in heavy hexagonal quantum codes, thereby increasing their reliability for practical quantum computing applications.

$\bullet$ **Humming sparrow optimization:** In the Humming Sparrow Optimization model, integrating herding and tracing characteristics orchestrates a dynamic exploration strategy. Sparrows, acting as producers, collectively scour the solution space for optimal solutions, embodying a herding behavior. The wealth of knowledge amassed during this phase is then shared with scroungers, individual sparrows that exhibit tracing behavior. Scroungers utilize this shared information to refine their search, concentrating on promising regions identified during the herding phase. This collaborative process, combining collective exploration and individual refinement, establishes a powerful synergy. The iterative exchange of insights between producers and scroungers propels the algorithm towards efficient convergence, enhancing its ability to discover optimal solutions across various optimization problems.

$\bullet$ **Self-adaptive Deep CNN Noise Correction Module** The Humming Sparrow Optimization method plays a pivotal role in fine-tuning the weights and biases of a classifier within a SADCNN model. Through the iterative application of the Humming Sparrow Optimization technique, the SADCNN adapts its parameters to enhance the accuracy.

The research demonstrates meticulous organization through distinct sections, each dedicated to addressing specific facets of the investigation. Section 2 meticulously pinpoints and elaborates on the limitations inherent in existing quantum error-correcting models. Transitioning to Section 3, the paper delves into the technical intricacies of the enhanced error-correcting model, offering a detailed exploration of its advancements. Section 4 succinctly encapsulates the experimental results derived from the proposed approach, providing a concise yet comprehensive overview of its empirical performance. Lastly, Section 5 serves as the culmination, presenting a conclusion summarizing the key findings and underscoring the significance of the error-correcting model in the broader context of quantum computing advancements.

## Literature review

Yoni Choukroun and Lior Wolf^1^ introduced a pioneering approach that effectively addresses the challenges posed by Quantum Error Correction Codes (QECC) constraints, offering a highly accurate solution for a diverse range of topological codes. Despite its success, the model is susceptible to overfitting and uniquely mitigates the measurement collapse phenomenon.

In a distinct realm, Hendrik Poulsen Nautrup et al.^2^ presented a reinforcement learning framework designed for optimizing and adapting quantum error correction codes in a fault-tolerant manner. This innovative approach allows the model to adapt to complex environments dynamically. However, training reinforcement learning agents for QECC involves a substantial requirement for training data.

Akshay Ajagekar et al.^3^ presented a fault diagnosis framework for analyzing faults in electrical power systems. The model utilizes unsupervised learning of the Conditional Restricted Boltzmann Machine (CRBM) network through quantum generative training with quantum sampling facilitated by an Adiabatic Quantum Computing (AQC) device. This method achieved superior fault detection and diagnosis performance yet faced limitations due to the relatively restricted accessibility of scalable quantum hardware.

Meng Cao^6^ contributed by presenting GRS codes and extended GRS codes, constructing eleven families of Maximum Distance Separable (MDS) codes with Galois hulls of arbitrary dimensions through various tools. This advancement significantly improved error correction capabilities, albeit with limited applicability.

Considering state-of-the-art error-correction codes compatible with locally connected 2D architectures, Bence Hetenyi et al.^7^ introduced four different qubit layouts essential for quantum memory experiments. These layouts effectively reduced the connectivity requirements for the surface code yet faced constraints related to the Error Threshold.

Changjun Kim et al.^8^ presented a quantum error mitigation scheme based on classical machine learning methods, enhancing the accuracy of Noisy Intermediate-Scale Quantum (NISQ) algorithms without extensive error characterization or quantum error correction. Despite its merits, this model encountered potential risks of overfitting.

Carlos Galindo et al.^9^ addressed the scenario where phase-shift errors are more likely than qudit-flip errors. Their error-correcting code leverages this asymmetry in quantum errors, specifically in the combined amplitude damping and dephasing channel. However, the model’s resource-intensive nature presents a challenge that needs to be considered in practical applications.

### Challenges

$\bullet$ Quantum computers inherently exhibit noise from factors such as decoherence and gate errors. A comprehensive understanding and characterization of these error sources are imperative for the precise training of error correction models in quantum computing^2^.

$\bullet$ The demand for large datasets is critical in training deep learning models effectively for quantum error correction. However, generating such datasets poses a significant challenge, particularly for fault-tolerant quantum error correction codes, given the computational expense of simulating quantum systems at scale^3^.

$\bullet$ Developing hybrid models that seamlessly integrate quantum error correction with classical deep learning components is a complex undertaking. Ensuring smooth integration and synchronization between the quantum and classical components presents a noteworthy challenge in the pursuit of effective error correction strategies^5^.

$\bullet$ Constructing accurate quantum error models replicating real quantum error patterns is challenging. The choice of an error model becomes crucial as it substantially influences the overall performance of error correction models in the quantum computing domain^6^.

$\bullet$ Ensuring the generalizability of error-correction models across diverse quantum systems and various error types is paramount. Achieving this level of generality proves to be a formidable task, demanding rigorous efforts in research and development to enhance the adaptability and robustness of quantum error correction methodologies^7^.

## Methodology for the proposed quantum error correction model using Humming sparrow optimization based Self-adaptive Deep CNN Noise Correction Module

**Figure 1:**
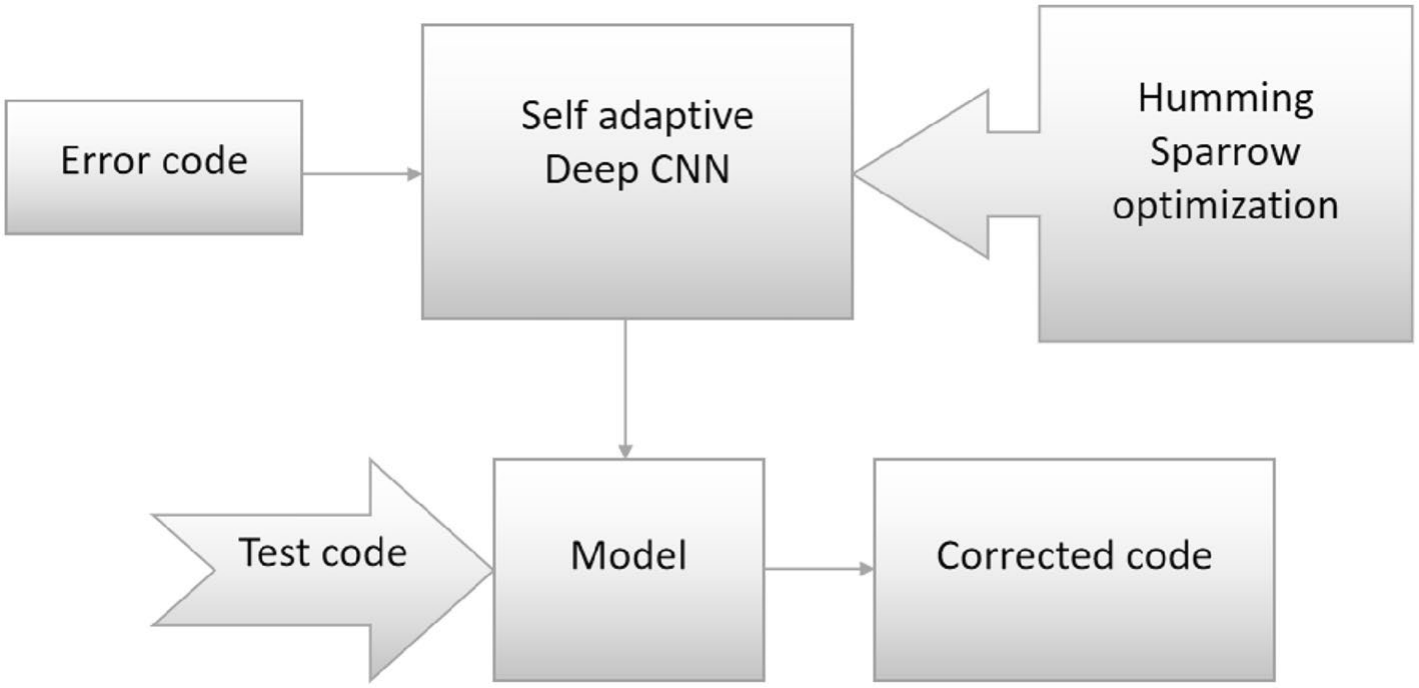
Self-adaptive noise correction module.

**Figure 2:**
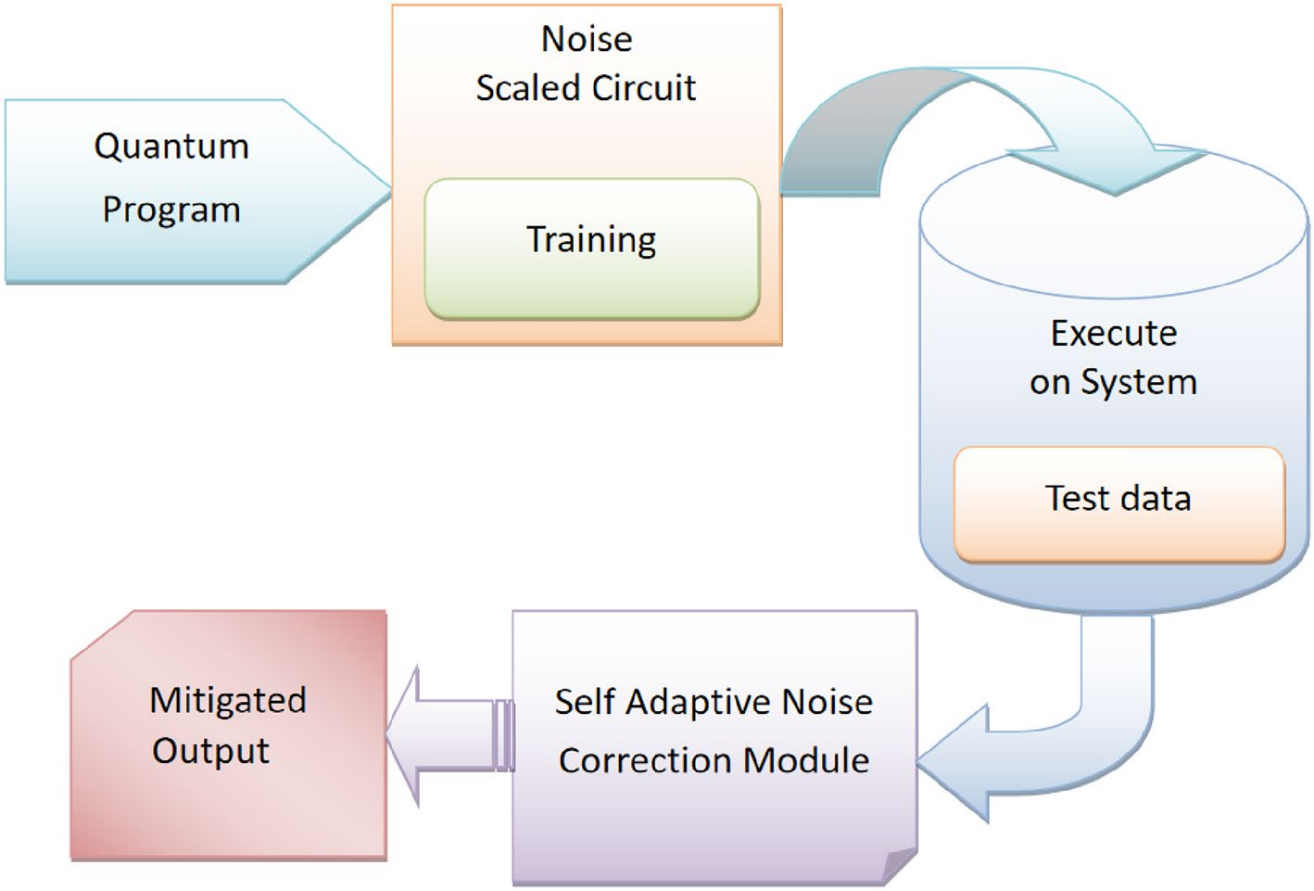
Architecture of the proposed quantum error correction module.

Quantum computing devices, despite their immense potential, are highly susceptible to noise due to unwanted interactions with their environment. The theory of quantum error correction provides a framework to mitigate the effects of noise on quantum states, thereby enabling robust and scalable quantum computers. In essence, quantum error correction model aims to preserve quantum information against decoherence and other errors. This research delves into the quantum error-correction domain, specifically focusing on topological quantum error-correcting codes tailored to safeguard against errors inherent in quantum computers employing superconducting qubits. The research introduces a novel approach to error correction for heavy hexagonal codes, a subclass of topological codes. Despite the existence of various decoding methods for topological codes, identifying the optimal decoder remains a formidable challenge. Addressing this challenge, the research leverages deep learning as a promising avenue. A dedicated deep learning-based decoder is proposed, tailored for heavy hexagonal codes and incorporating a SADCNN module, as illustrated in Fig. 1. The SADCNN module utilizes a CNN architecture which is designed to process quantum states as input. It learns to identify patterns associated with noise and correct errors. The CNN adaptively adjusts its parameters during training using self adaptive mechanism which adapts to the specific characteristics of error codes generated by heavy hexagonal codes. This adaptability is crucial for effectively addressing the challenges posed by errors in quantum computations. The effectiveness of the proposed decoder on heavy hexagonal codes is systematically evaluated across different code distances three, five, and seven through the utilization of the HSO algorithm. HSO is crafted to fine-tune the SADCNN decoder, enhancing its ability to correct errors in heavy hexagonal quantum codes and thereby bolstering their reliability for quantum computing applications. This algorithm amalgamates the advantageous characteristics of Humming Bird optimization^14^ and Sparrow search optimization^15^. The schematic representation of this innovative methodology is elucidated in Fig. 2, showcasing the intricate interplay of the proposed components in the pursuit of more robust and accurate quantum error correction.

### Overview of heavy hexagonal codes

Heavy hexagonal codes represent a specialized subclass of topological quantum error-correcting codes uniquely tailored for quantum computing architectures employing superconducting qubits. These codes leverage the distinctive geometry of hexagonal lattice structures to encode logical qubits. The topological nature of heavy hexagonal codes allows for fault-tolerant quantum computation by exploiting the spatial relationships among physical qubits. Through this approach, heavy hexagonal codes offer inherent resilience against certain types of errors, providing a robust foundation for quantum information storage and processing. Heavy hexagonal codes play a pivotal role in quantum error correction due to their specific attributes. The topological protection afforded by the hexagonal lattice structure enables these codes to guard against errors resulting from local disturbances and imperfections in the quantum hardware. This inherent fault tolerance is crucial for enhancing the stability of quantum computations, making heavy hexagonal codes particularly well-suited for architectures utilizing superconducting qubits. Additionally, the scalability and adaptability of heavy hexagonal codes contribute to their relevance, allowing for the construction of larger quantum circuits while accommodating variations in qubit connectivity and interaction strengths. Overall, heavy hexagonal codes represent a promising avenue in quantum error correction, addressing challenges associated with the reliability and efficiency of quantum algorithms in superconducting qubit-based quantum computers.

**Background**

**Classical Error Correction Code** In the realm of error-correcting codes (ECC), a linear code *L* is precisely characterized by a binary generator matrix *M* of dimensions $t\times i$ and a binary parity check matrix *T* of dimensions $(i-t)\times i$. The matrices are chosen in such a way that their product $GH^{T}$ results in the zero over the order-2 Galois field$(GF_{2})$.

When encoding the input message $n\in \{0,1\}^{t}$ using the generator matrix *G*, a code word is generated $y\in D\subset \{0,1\}^{t}$. This code word satisfies the condition $Hy=0$ and is subsequently transmitted through a symmetric channel, such as an Additive White Gaussian $Nois_{2e (AWGN)}$ channel. Denoting the channel output as *x*, expressed as $x= y_{u} +\epsilon \subset S \in {\mathcal {R}}^{i}$ where $y_{u}$ represents the modulation of *y* (Binary Phase Shift Keying - BPSK), and $\epsilon$ is random noise independent of the transmitted *y*. The primary objective of the decoder $f: S\rightarrow \{0,1\}^{i}$ is to provide an accurate approximation of the original codeword $y = f(x)$.

In ECC, the important notation is the syndrome which is obtained by the binary mapping multiplication of *x* with the parity check matrix over$GF_{2}$ such as

1 $$\begin{aligned} u = u(x) = Hx_{e} = H(y\oplus \epsilon _{e}) = H\epsilon _{e} \end{aligned}$$

Consider the XOR operator denoted by $\oplus$ where $x_{e}$ and $\epsilon _{e}$ represent the hard-decision vectors of *x* and $\epsilon$, respectively.

**Quantum Error Correction Code** The transition from classical bits to quantum bits (qubits) characterizes the pivotal shift into the quantum realm. The quantum state of a qubit, denoted as $|\psi \rangle$, is defined by

2 $$\begin{aligned} |\psi \rangle = \alpha |0\rangle + \beta |1\rangle s.t. \alpha ,\beta \in {\mathbb {C}}, |\alpha |^2 +|\beta |^2 =1 \end{aligned}$$

A coherent quantum error process, denoted as *A*, can be dissected into a summation of operators drawn from the Pauli set $\{I, X, Z, XZ\}$. In this context, the Pauli basis is characterized by the identity mapping *I*, the quantum bit-flip *X*, and the phase-flip *Z*. This decomposition allows the characterization of single-qubit errors as

$$I|\psi \rangle = |\psi \rangle$$

$$X|\psi \rangle = \alpha X |0\rangle + \beta X |1\rangle = \alpha |1\rangle + \beta |0\rangle$$

$$Z|\psi \rangle = \alpha Z |0\rangle + \beta Z |1\rangle = \alpha |0\rangle - \beta |1\rangle$$

3 $$\begin{aligned} A|\psi \rangle = \alpha _{I}|\psi \rangle + \alpha _{X}X|\psi \rangle + \alpha _{Z}Z|\psi \rangle + \alpha _{XZ}XZ|\psi \rangle \end{aligned}$$

With $\alpha _{I}|,\alpha _{X},\alpha _{Z},\alpha _{XZ}$ representing the expansion coefficients of the noise process, the no-cloning theorem establishes that a quantum state $|\psi \rangle$cannot be redundantly copied$(|\psi \rangle \otimes |\psi \rangle ,\dots ,\otimes \Vert \psi \rangle )$, where $\otimes$ denotes the n-fold tensor product. Despite this limitation, quantum information redundancy can be achieved through the logical state e, encoding of a given state $|\psi \rangle _{n}|$via quantum entanglement and a unitary operator *U*, such that $|\psi \rangle _{n} = U(|\psi \rangle \otimes |\psi \rangle ,\dots ,\otimes \Vert \psi \rangle )$. An illustrative example of such a unitary operator is the GHZ state, generated using CNOT gates. In this encoding, the logical state is defined within a subspace of the expanded Hilbert space. This subspace determines both the code space *L* and its orthogonal error space *F*, such that any error $A|\psi _{i} \in F$. The orthogonality of *L* and *F* enables the determination of the subspace occupied by the logical qubit through projective measurement, preserving the encoded quantum information. In the realm of quantum coding, the set $\rho$ comprises non-destructive measurements known as stabilizer measurements. These measurements are performed through additional qubits (ancilla bits). The collective result of all stabilizer measurements on a given state is termed the syndrome. For a given stabilizer generator $\rho E|\psi \rangle _{i} = |\psi \rangle _{i}$ results in an anti-commuting (-1 Eigen value) and thus detects an error *A*. In cases where the syndrome measurement is unreliable, it may be necessary to repeat the measure to enhance confidence in the outcome.

A significant category within the realm of Pauli operators is logical operators. Unlike elements of the stabilizer group, logical operators do not belong to the stabilizer group but exhibit commutativity with every stabilizer. While stabilizer operators act trivially in the code space $P|\psi \rangle _{i} = |\psi \rangle _{i}$, logical operators $L\in \zeta$ act non-trivially within it, introducing errors $E|\psi \rangle _{i} \in C s.t. L|\psi \rangle _{i} = |\psi \rangle _{i}$. These operators maintain commutation with stabilizers but can potentially represent undetectable errors. Hence, benchmarks for QECC commonly employ logical error metrics. These metrics gauge the disparity between the predicted projected noise $L_{\epsilon }$ and $L_{\epsilon }$ the actual noise, where *L* signifies the discrete matrix associated with the logical operator. This approach is integral for assessing the efficacy of quantum error correction, emphasizing the importance of capturing errors that may not be detected by stabilizer operators in the code space.

**QECC from the ECC perspective** An alternative approach to representing stabilizer codes involves decomposing the stabilizer operators into two distinct parity check matrices, thus defining the block parity check matrix $$\begin{bmatrix} H_{R} &{} |&{} 0\\ \hline 0 &{}|&{} H_{P} \end{bmatrix}$$. This matrix separates phase-flip checks $H_{P}$, and bit-flip fits $H_{R}$. The syndrome, denoted as *S*, is then computed through the multiplication of the encoded state ${\mathcal {U}} = N\epsilon$ with the check matrix *N*, incorporating the code stabilizers in $H_{R}$ and $H_{P}$, and the binary noise vector $\epsilon$. The primary objective of the quantum decoder $f: \{0,1\}^{|\rho |}\rightarrow \{0,1\}^{|\zeta |}$ is to provide an approximation of the noise, relying solely on the information conveyed by the syndrome. This process emphasizes the critical role of decoding in quantum error correction, where the decoder interprets the syndrome to infer and mitigate the effects of noise on the encoded quantum information.

Hence, the quantum scenario can be mapped onto its classical counterpart as follows: the *t* logical qubits resemble classical *t* information bits, and the *i* physical qubits parallel classical code words. Computation or simulation of the quantum state’s syndrome mirrors classical approaches, achieved by defining a binary parity check matrix constructed from the quantum code stabilizers. Noteworthy distinctions from classical ECC include (i) the absence of direct access to the current state, a capability standard in the classical domain where arbitrary measurement of *y* is routine; (ii) a focus on the logical qubits, anticipating the code up to the mapping introduced by logical operators *L* and finally, (iii) the need for repetitive sampling of the syndrome due to potential errors in syndrome measurements. The primary objective of the approach is to develop a decoder that is parameterized by a vector of weights, denoted as, to minimize the noise vector *e* in the equation $\epsilon = f_{\theta }(u)$. This decoder learning process is pivotal for effectively correcting errors in quantum information, accounting for the challenges introduced by quantum principles.

### Topological code

Topological quantum error-correcting codes represent the best approach to address the challenges posed by errors in quantum information processing. Among these, the surface code stands out as a prominent example. Topological codes leverage the principles of quantum topology to encode qubits in a manner that makes error detection and correction highly resilient. In the case of the surface code, qubits are arranged on a two-dimensional lattice, and logical qubits are encoded by non-contractible loops in this lattice. Errors typically result from environmental noise or imperfections in quantum gates, manifest as defects or excitations within the lattice. The topological approach lies in its ability to detect and correct errors by identifying changes in the global properties of the encoded state rather than relying on local measurements. Consequently, topological codes offer a robust foundation for building fault-tolerant quantum computers, with the surface code being a particularly promising candidate due to its simplicity and effectiveness in error mitigation.

#### Encoding and decoding of surface code

The encoding and decoding processes in the surface code are fundamental to its efficacy as a topological quantum error-correcting code. During the encoding phase, logical qubits are mapped onto physical qubits arranged on a two-dimensional lattice. Specifically, information is encoded by creating non-contractible loops or plaquettes in the lattice. The entanglement of qubits in these loops forms the basis for quantum error protection. When errors occur due to noise or other disturbances, the surface code’s ingenious design enables the identification of errors through syndrome measurements. Decoding involves interpreting these syndromes to pinpoint and correct errors without directly measuring the state of individual qubits. The global nature of these topological measurements allows for fault-tolerant error correction, as only the global properties of the encoded state are analyzed. This robust approach distinguishes the surface code and other topological codes from traditional error correction methods, making them promising for building reliable quantum computers.

#### Surface code and lattice representation diagram of the surface code

**Figure 3:**
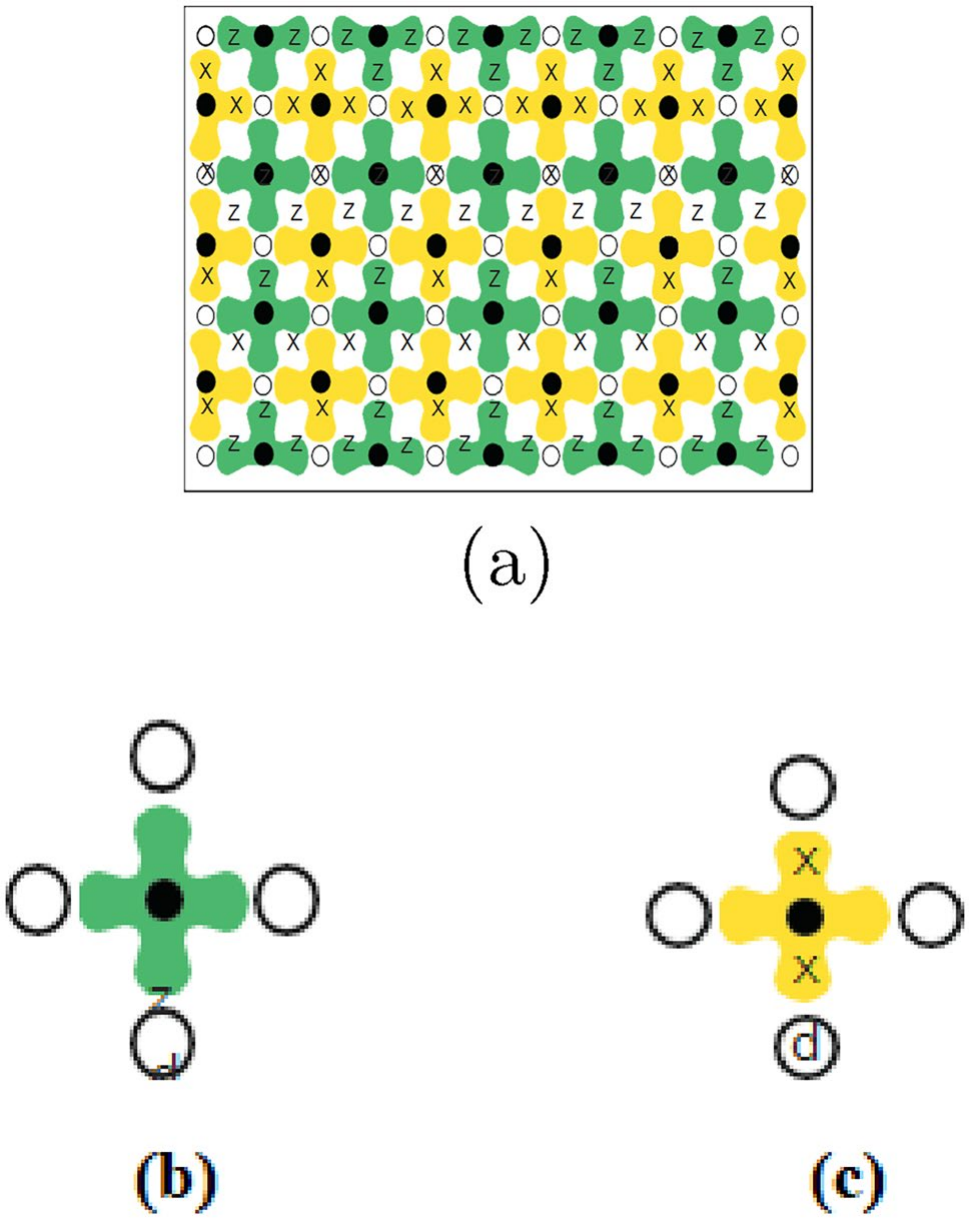
Lattice representation diagram of the surface code.

The example of a two-qubit stabilizer offers a glimpse into a rudimentary form of error detection. However, more intricate circuits can provide error detection and precise identification in significantly larger qubit assemblies, exemplified by the surface code. In implementing the surface code, utilize a two-dimensional array of physical qubits, as depicted in Fig. 3. The qubits fall into two categories: data qubits, represented by open circles in Fig. 3a, where computational quantum states are stored, and measurement qubits, represented by solid circles. Both data and measurement qubits must meet the fundamental requirements for quantum computation, including initialization, single-qubit rotations, and a two-qubit CNOT operation between nearest neighbors. Additionally, to perform a topological version of the Hadamard transformation, the data qubits and measurement qubits must be capable of exchanging their quantum states through a SWAP operation. A method for measuring ${\hat{Z}}$ for each qubit is also essential. The measurement qubits play a crucial role in stabilizing and manipulating the quantum state of the data qubits. There are two types of measurement qubits: measure-*Z* qubits, colored green (dark) in Fig. 3a, and measure-X qubits, colored orange (light). These are referred to as Z syndrome and X syndrome qubits, respectively, in the surface code. Each data qubit is coupled to two measure-*Z* qubits and two measure-*X* qubits, while each measurement qubit is coupled to four data qubits. A measure-*Z* qubit compels its neighboring data qubits *a*, *s*, *c*, and *d* into an Eigen state of the operator product ${\hat{Z}}_{a},{\hat{Z}}_{b},{\hat{Z}}_{c},{\hat{Z}}_{d}$. Consequently, each measure-*Z* qubit measures a ${\hat{Z}}$ stabilizer. Similarly, a measure-*X* qubit forces its neighboring data qubits into an Eigen state of ${\hat{X}}_{a},{\hat{X}}_{b},{\hat{X}}_{c},{\hat{X}}_{d}$ thereby measuring an *X* stabilizer. This intricate arrangement forms the foundation of the surface code, enabling robust error detection and correction in quantum computations.

Figure 3a depicts the illustration of the two-dimensional array implementation of the surface code. Data qubits are denoted by open circles $(\circ )$, and measurement qubits are represented by solid circles $(\bullet )$, with measure-*Z* qubits in green (dark) and measure-*X* qubits in orange (light). In the interior, each data qubit interacts with four measure qubits, while each measure qubit contacts four data qubits, performing four-terminal measurements. At the boundaries, measure qubits interact with only three data qubits, conducting three-terminal measurements, and data qubits connect with either two or three measure qubits. The solid line demarcating the array outlines its boundary. Figure 3b is the depiction of the geometric sequence of operations (left) and the corresponding quantum circuit (right) for one surface code cycle involving a measure-*Z* qubit. This process stabilizes ${\hat{Z}}_{a},{\hat{Z}}_{b},{\hat{Z}}_{c},{\hat{Z}}_{d}$. Figure 3c is the configuration and quantum circuit for a measure-*X* qubit, responsible for stabilizing ${\hat{X}}_{a},{\hat{X}}_{b},{\hat{X}}_{c},{\hat{X}}_{d}$. The two identity operators ${\hat{I}}$, which signify waiting periods, are incorporated in the measure-*Z* process, aligning the timing with that of the measure-*X* qubit. The latter undergoes two Hadamard ${\hat{H}}$ operations, with the identity operators positioned at the sequence’s outset and conclusion to minimize the impact of errors during these crucial steps.

**Error correction model** Surface codes and Bacon Shor codes are the most promising error-correcting codes for building quantum computers. Stabilizer formalism is an important basis for understanding the heavy hexagonal code. The hexagonal lattice code is a quantum error-correcting code that maps *t* logical qubits to *i* physical qubits and uses a $2^{t}$ dimensional code space $H_{c}$ to accommodate these encoded qubits. The code space can be viewed as a $2^{t}$ -dimensional subspace of the $2^{i}$ -dimensional Hilbert space defined by code words $|\psi \rangle$

4 $$\begin{aligned} H_{c} = \{|\psi \rangle \in H_{c}|\psi \rangle = |\psi \rangle \forall c\in H \} \end{aligned}$$

The stabilizer group, *H*, is an Abelian subgroup consisting of the Pauli group *M* of *i* qubits. *Z*(*H*) is the set of Pauli group elements that satisfy $Z(H) = \{M \in M_{i} | MH = HM | \forall H \in H\}$. The stabilizer code $D_{H}$ is composed of the minimum number of *p* generators $H_{q}$.

5 $$\begin{aligned} H = \langle H_{1},H_{2},\dots , H_{p} \end{aligned}\rangle$$

And each encoded qubit has a pair of logical operators $\bar{X_{q}}, \bar{Z_{q}}$,

6 $$\begin{aligned} \bar{X_{q}}, \bar{Z_{q}} \in X(H)-H; \bar{X_{q}} \bar{Z_{q}} = - \bar{Z_{q}} \bar{X_{q}}, \end{aligned}$$

Then, perform a stabilizer measurement for each generator $H_{q}$ in *H* and define the syndrome vector $h \in Z_{2}^{p}$ as

7 $$\begin{aligned} H = (H_{1},H_{2}, \dots ,H_{p}) \text{ where } H_{q} = {\left\{ \begin{array}{ll} 0, &{} {\textbf {if}} \text{ measure of } H_{q} \text{ yields } +1\\ 1, &{} {\textbf {otherwise}} \end{array}\right. } \end{aligned}$$

In this research, we develop a novel quantum error correction approach by combining elements from surface codes and Bacon Shor codes to enhance the error correction properties of the heavy hexagonal code. The heavy hexagonal code exhibits characteristics of both surface codes and Bacon Shor codes, allowing us to leverage their respective error correction mechanisms. For error correction in the heavy hexagonal code, adopt a strategy where X and Z errors are addressed through the decoding processes of surface codes and Bacon Shor codes, respectively. Specifically, X errors are corrected using classical topological surface code decoding, while Z errors are rectified through the Bacon Shor code decoding process. This dual approach capitalizes on the strengths of both codes to effectively correct errors in the heavy hexagonal code. CNOT gates, widely used in quantum computing for qubit interactions, play a crucial role in the approach. Here focus is on optimizing the CNOT gate operation order to minimize the overall number of error positions during the synthesis measurement. Our scheduling algorithm aims to streamline the sequence of CNOT gate operations, thereby enhancing the error correction efficiency of the heavy hexagonal code. In the heavy hexagonal code, error correction involves an 11-time-step cycle (7 for X errors and 4 for Z errors), encompassing qubit initialization and measurement. Despite having more complex measurement circuits compared to topological surface codes, the heavy hexagonal code gains a significant advantage by employing flag qubits. These flag qubits enable the correction of weight-two errors arising from a single error during measurements of the weight-four X gauge. To evaluate the error correction performance of the heavy hexagonal code under simplified noise models, here integrate the depolarization noise model into our analysis. The depolarization noise model is processed independently, simplifying the noise characterization for the experiments. Given a quantum state, the evolution of the state in the depolarization noise model is carefully considered and incorporated into the evaluation framework. Through this innovative approach, we aim to enhance the error correction capabilities of the heavy hexagonal code and contribute valuable insights to the field of quantum computing

8 $$\begin{aligned} \rho \rightarrow (1-\rho _{x}-\rho _{y}-\rho _{z})\rho + \rho _{x}X_{\rho X^{+} + \rho _{y}Y_{\rho } Y^{+}} + \rho _{z}Z_{\rho }Z^{+} \end{aligned}$$

**Figure 4:**
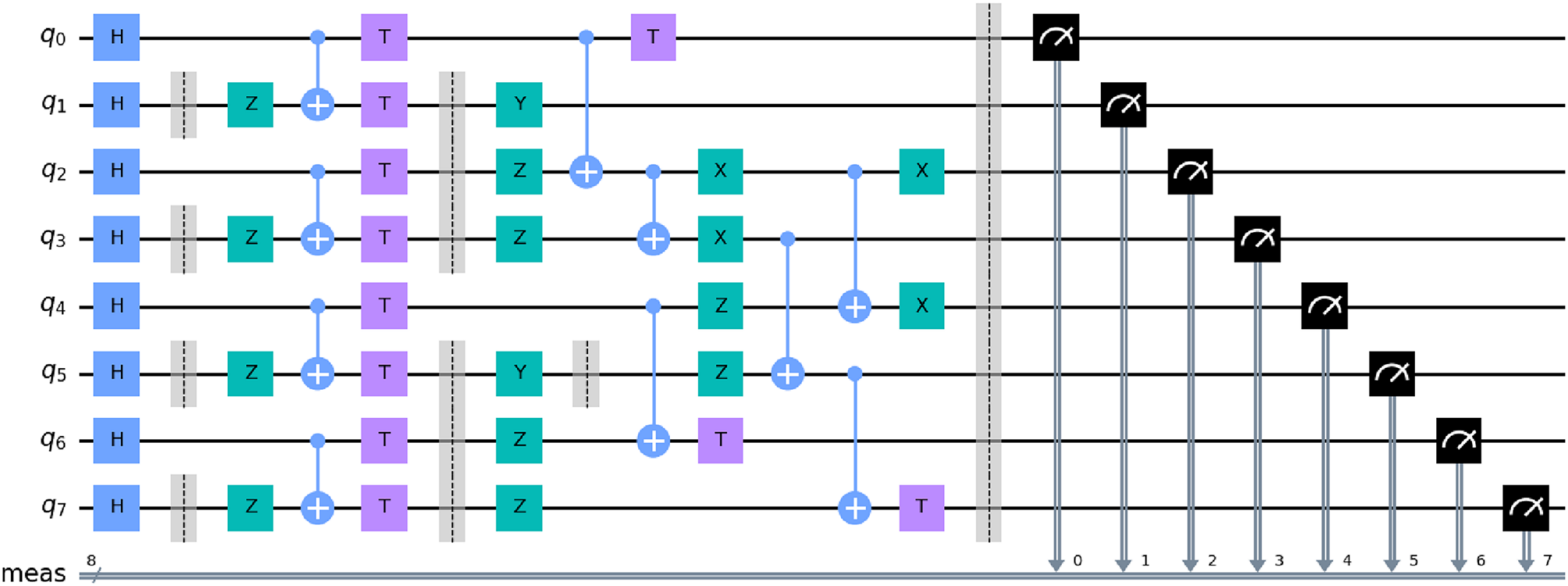
Architecture of the quantum circuit.

And $\rho _{x} = \rho _{y} = \rho _{z}$ In the heavy hexagonal code measurement circuit, where $M_{x}, M_{y}$ and $M_{z}$, denoting the probabilities of Pauli *X*, *Y* and *Z* errors , respectively, a flag qubit is considered flagged upon obtaining a nontrivial measurement result. Nontrivial flag outcomes signify occurrences of weight-two or weight-four data qubit errors or instances where a measurement error triggered the flag. The measurement syndrome is characterized by the flag qubit exhibiting nontrivial results in *X* type gauge operator measurements during *X* errors on qubits and the measurement of erroneous data qubits during *Z* stabilizer measurements. Similar to *X* errors, the *X* error captures the measurement syndrome of the data qubit by interpreting the flag qubit’s measurement outcomes. This process enables the inference of the data qubit where the error occurred. The measurement circuit is iteratively looped to extract syndrome information, which is subsequently analyzed to correct errors that transpired on the qubits. Given the complexity of combining syndrome information, conventional statistical methods face challenges in their selection. Consequently, we propose a self-adaptive Deep CNN noise correction module that leverages the translation invariance of the heavy hexagonal code to input error syndromes into a CNN, enabling a more rapid determination of optimal syndromes. The goal is to achieve the maximum threshold for error correction by optimizing conditions. This research places emphasis on exploring the local correlation of error syndromes based on the distance of the heavy hexagonal code. Utilizing a self-adaptive Deep CNN aims to exploit the inherent features and relationships within syndromes to enhance the accuracy and efficiency of error correction. This approach allows for adaptive learning, enabling the model to discern patterns in syndromes and make informed decisions in correcting errors. Focusing on the degree of local correlation aims to uncover the spatial dependencies among error syndromes, which can provide valuable insights into optimizing the performance of the heavy hexagonal code under varied conditions. The self-adaptive nature of the proposed Deep CNN module ensures versatility and adaptability in addressing the challenges posed by the intricacies of syndrome information in quantum error correction. Quantum circuits are a sequence of matrix operations performed on qubits, the quantum version of a bit. A quantum circuit takes some qubits as input, it performs matrix operations on them, and then as output we take measurements and get a particular state vector. The architecture of a quantum circuit is illustrated in Fig. 4,

## Working of the SADCNN Noise Correction Module in quantum error correction

**Figure 5:**
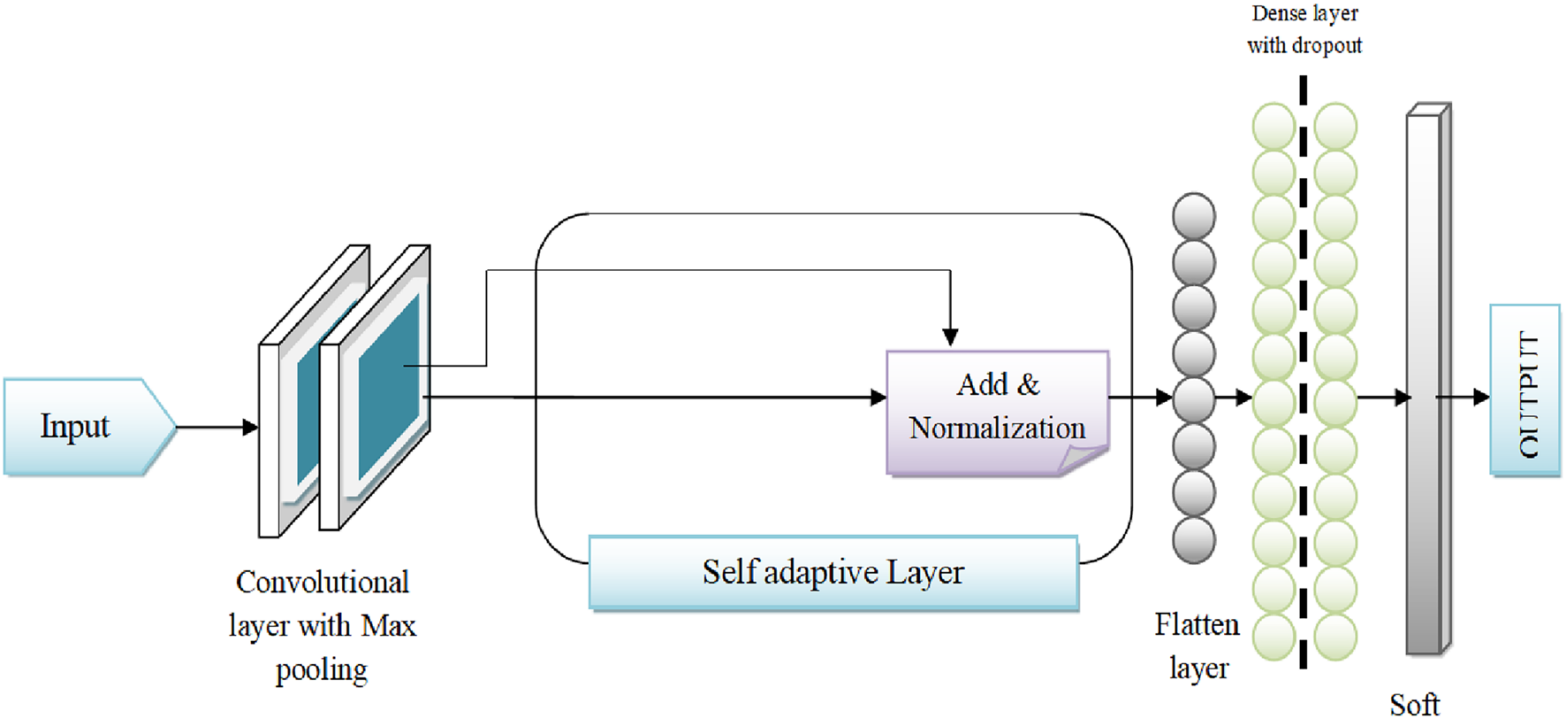
Architecture for the SADCNN Noise Correction Module in quantum error correction.

**Figure 6:**
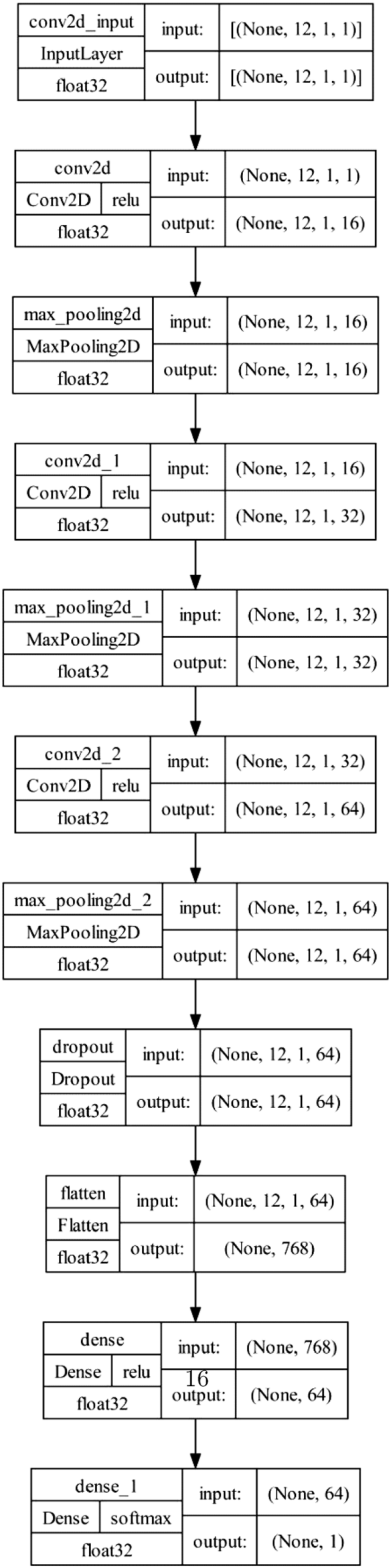
Layer details for the SADCNN Noise Correction Module in quantum error correction.

At the core of the proposed decoder is the SADCNN noise correction module, which is meticulously designed to play a pivotal role in mitigating noise and errors within the quantum data associated with heavy hexagonal codes. Its primary objective is to enhance the decoding process by effectively addressing the challenges posed by noisy quantum channels. The architecture of the SAD- CNN Noise Correction Module is characterized by its adaptability, leveraging deep Convolutional neural networks (CNN). SADCNN extracts intricate features from the noisy quantum data. These features play a crucial role in identifying and correcting errors. The modules functionalities extend beyond conventional noise correction, incorporating self-adaptive mechanisms that dynamically adjust to the evolving error patterns in heavy hexagonal codes. This adaptability ensures a robust response to errors dynamic and multifaceted nature, contributing significantly to the decoders overall performance. By effectively correcting errors, it enhances the reliability of decoded quantum information. Its self-adaptive nature allows it to respond dynamically to varying error scenarios. Whether due to environmental fluctuations or inherent quantum noise, SADCNN remains resilient. The SADCNN Noise Correction Module in the proposed quantum error correction methodology are designed to enhance the decoding process for heavy hexagonal codes. The SADCNN leverages deep learning techniques and adapts its structure to correct errors in quantum codes effectively. The architecture for the SADCNN Noise Correction Module in quantum error correction is illustrated in Fig. 5. In Fig. 6, it shows the Layer details for the SADCNN Noise Correction Module. Lets delve into the workings of the SADCNN module.

$\bullet$**Input Layer** The input layer in a quantum error-correcting neural network receives quantum error codes as its input. These codes represent the quantum state, potentially corrupted by errors. The layer’s role is to establish the initial representation of the encoded quantum information, creating a foundation for subsequent processing. Here, the error code input is denoted as *X*.

$\bullet$ **Convolutional Neural Network Architecture Based Decoder** A CNN is a class of artificial neural networks commonly employed for tasks involving data classification in machine learning. This deep learning algorithm is primarily constituted by three key components: convolution, pooling, and activation. The initial layers of the CNN consist of Convolutional layers, these layers use filters (kernels) to convolve over the input error code tensor, extracting features that are relevant for identifying specific error patterns. Activation functions, such as ReLU (Rectified Linear Unit), intro- duce non-linearity to the model. This allows the CNN to learn complex relationships and patterns within the error codes and these layers learn spatial hierarchies of features from the noisy data. The convolution layer $(Y_{conv1},Y_{conv2}, \dots )$capture hierarchical features from the input:

9 $$\begin{aligned} Y_{conv} = \sigma (W_{conv}*X + b_{conv}) \end{aligned}$$

Where, $W_{conv}$ are the weights, $b_{conv}$ is the bias, and $\sigma$ is the activation function.

$\bullet$ **Pooling layer:** The layers within the POOL mechanism aim to reduce the spatial dimensions of feature maps derived from the Convolutional layers through down-sampling techniques. This approach prioritizes crucial features, effectively lowering computational complexity and spatial volume. Max pooling operation can be represented as:

10 $$\begin{aligned} Y_{pool} = Maxpool(Y_{conv}). \end{aligned}$$

$\bullet$ **Self-Adaptive Mechanism** A self-adaptive deep CNN for quantum error correction is designed to dynamically adjust its architecture, learning rates, weights, and other parameters to adapt to the specific characteristics of error codes generated by heavy hexagonal codes. This adaptability is crucial for effectively addressing the challenges posed by errors in quantum computations. The self-adaptive nature of the SADCNN module is of paramount importance, signifying that the CNN’s architecture dynamically adjusts throughout the training process based on the input characteristics. This adaptability empowers the network to tailor its structure to heavy hexagonal quantum codes’ distinct features and complexities. Embedded within the SADCNN is a dedicated Noise Correction Module, enhancing the decoded information by applying corrections to syndromes and effectively mitigating errors introduced during quantum computations.

11 $$\begin{aligned} Y_{adaptive} = \text{ adaptive normalization }(Y_{pool}) \end{aligned}$$

$\bullet$ **Dense Layer** The dense layer plays a critical role in further processing the features, which consists of densely connected neurons, allowing the network to capture intricate relationships and dependencies within the quantum information. This layer is pivotal for refining the representation of the quantum states and preparing the data for the final error correction steps. Fully connected layer with weights *W*, bias *b*, and activation function $\sigma$:

12 $$\begin{aligned} Y_{Dense} = \sigma (W_{Dense}Y_{Dense} + b_{Dense}) \end{aligned}$$

$\bullet$ **Output layer** The output layer is responsible for producing the corrected quantum states, which employ a quantum-appropriate activation function, which could be a variant of the softmax function designed to suit quantum computations. The layer outputs a probability distribution over the possible quantum states, with the goal of selecting the corrected state with the highest likelihood. This final layer completes the quantum error correction process within the neural network framework.

13 $$\begin{aligned} S(Y_{Dense}) = \frac{e^{yi}}{\sum _{j} e^{yi}} \end{aligned}$$

Here, $y_{i}$ denotes the raw output of the $i^{th}$ unit in the dense layer before applying the softmax function. These logits represent the unnormalized scores associated with each class. The symbol $\sum _{j}$ denotes the summation operation, where *j* iterates over all the classes. This summation is performed in the denominator to normalize the exponentials. The SADCNN Noise Correction Module combines the power of deep learning with adaptability, making it an indispensable asset in quantum error correction. Its ability to dynamically adjust to evolving error patterns ensures reliable decoding even in challenging quantum environments.

## Proposed humming sparrow optimization

HSO excels in dynamically adjusting parameters, aligning with the self-adaptive nature of the SADCNN Decoder, which intelligently tunes the weights and biases of the decoder, adapting them to the specific error patterns encountered during the decod- ing process. This adaptability is vital for optimizing the performance of the SADCNN module across different code distances. It is tailored to address computationally intensive tasks in high-performance computing scenarios. The Humming Sparrow Optimization model represents a novel approach inspired by the collective foraging behaviors of sparrows. In this model, sparrows act as explorers and producers, dynamically navigating a search space for optimal food sources. The optimization process involves a synergistic blend of herding and tracing characteristics. During the herding phase, sparrows collectively explore potential food-rich areas, mimicking the collaborative foraging observed in nature. Subsequently, in the tracing phase, individual sparrows utilize the shared information from the exploration phase to refine their search, focusing on promising regions. The crucial aspect lies in the seamless exchange of information, where the producers transmit their findings to the scroungers. This collaborative knowledge-sharing mechanism facilitates a more informed and efficient convergence towards optimal solutions. By emulating the combined herding and tracing behaviors, the Humming Sparrow Optimization model aims to enhance convergence speed and solution quality in optimization problems, drawing inspiration from the cooperative dynamics observed in sparrow communities.

$\bullet$ **Inspiration** The hybrid sparrow and hummingbird optimization algorithm draws inspiration from the adaptability of sparrows and the efficiency of hummingbirds in nature. This synergy is harnessed to address complex optimization problems, where the algorithm dynamically adapts like sparrows to diverse environments and explores with precision similar to hummingbirds. The approach strikes a balance between adaptability and efficiency, creating a symbiotic collaboration that allows the algorithm to gracefully navigate through intricate optimization landscapes. The visual metaphor of a flock in flight captures the essence of this algorithm, orchestrating an optimization symphony where adaptability and precision work in tandem to achieve optimal solutions in diverse and dynamic problem spaces.

$\bullet$ **Initialization** The initial positioning of the searcher within the search space serves as a critical parameter in the optimization algorithm, which establishes the starting point for the exploration process and significantly influences the algorithm’s ability to find optimal solutions.

14 $$\begin{aligned} X_{t+1} = X_{t} - rand(u-l) + X_{g}^{t-1}(u-l) + X_{p}^{t-1}(u-l) \end{aligned}$$

Where, $X_{p}^{t-1}$ denotes the personal best at $t-1$ iteration, $X_{g}^{t-1}$ denotes the global best at $t-1$ iteration, $X_{t}$ denotes the current position, denotes the upper bound, *l* denotes the lower bound and *rand* range is from (0, 1) At t= 1 the equation becomes

15 $$\begin{aligned} X_{t+1} = X_{t} rand(u-l) +2(u-l). \end{aligned}$$

$\bullet$ **Evaluate the fitness** The optimal fitness of a solution is determined by evaluating its performance against the objective function in the context of tuning a SADCNN model. Specifically, how accurately the SADCNN model corrects errors The most favorable solution for enhancing the SADCNN model is characterized by achieving the highest accuracy in error correction. This evaluation step guides the optimization process by providing feedback on the quality of the current solution.

$$\bullet$$ **Case 1** if $$X^{t+1} > fit(X_{q}^{t})$$

$\bullet$ **Leading phase** In this phase, the searcher autonomously explores the search space in pursuit of optimal solutions, a characteristic fundamental to the effectiveness of optimization algorithms, which representing an individual or agent within the algorithm, engages in independent search efforts. This autonomous exploration is crucial for achieving increased convergence, as it allows the algorithm to navigate the solution space dynamically and adapt to the evolving landscape of potential solutions. The searcher’s ability to explore the search space independently enables a more comprehensive coverage of diverse regions, facilitating the discovery of promising areas that contribute to improved convergence, which empowers the algorithm to efficiently converge towards optimal solutions without relying on external directives.

$$\bullet$$ **condition-1** if $$fit(X^{t+1} = fit(X^{t}) )$$ & $$X^{t+1} > fit(X_{q}^{t})$$

$\bullet$ **Exploitation Phase** At this condition, when the current and updated position of the searcher remains the same, the Humming Sparrow Optimization model employs a Levy flight strategy for food searching. This strategy is employed to introduce a form of random exploration when the algorithm encounters stagnation or lack of improvement in fitness values and the introduction of levy flights adds a stochastic element to the algorithm helping it escape local optima and explore new regions of the solution space. A Levy flight is a type of random walk where the step lengths are drawn from a probability distribution known as the Levy distribution. By incorporating Levy flights in such situations, the algorithm introduces a degree of randomness, allowing it to escape local optima and explore new regions of the solution space. This stochastic element can be especially beneficial in enhancing the algorithm’s ability to discover more diverse and potentially better solutions, even when faced with fitness plateaus or stagnation in the optimization process. The Levy flight mechanism adds an element of exploration to the algorithm, contributing to its adaptability and robustness in navigating complex solution landscapes. By introducing randomness into the search process, the algorithm becomes more resilient to changes in the environment and better able to handle diverse optimization scenarios

16 $$\begin{aligned} X^{t+1} = X^{t} + \alpha _{1} \otimes levy(\beta ) \end{aligned}$$

$$levy(\beta ) = \mu l|\nu |^{\frac{1}{\beta }}$$

$\alpha = \alpha _{0}(X^{t} - X_{p}^{t}) \alpha _{0}$ denotes the constant

$$\mu = \frac{1}{\sigma _{\mu }^{\sqrt{2\pi }}} e^{\frac{\lfloor (X_{t-1} -X_{t})_{\mu }\rfloor }{2\sigma _{\mu }^{2}}}$$

Here, $\sigma$ denotes the standard deviation, $\mu$ denotes the variance and $X_{t}$ describes the mean of $X_{t}$ until iteration *t* where , $\sigma _{\mu } = \frac{\Gamma (1+\beta ) sin(\pi \beta /2)}{[\Gamma (1+\beta /2)]_{\beta 2^{\frac{\beta -1}{2}}}}$, $\beta = 1.5$ and $\Gamma (1+\beta ) = \int _{t_{0}}^{t_{max}} e^{-t} t^{\beta } dt$ Here,$t_{0}$ denotes the initial iteration, $t_{max}$ denotes the maximum iteration

$$\text{V} = \frac{t_{max -\mu }}{\sigma _{\mu }}$$

$\bullet$ **condition-2** if $fit(X^{t+1}) > fit(X^{t})$ & $(X^{t+1}>fit(X_{g}^{t}))$ If this condition is satisfied, this means that the fitness at $X^{t+1}$ changes from $X^{t}$ iteration

17 $$\begin{aligned} X^{t+1} = X^{t} + r(X^{t} - X^{t-1}) \end{aligned}$$

Where, $r = cos(\theta _{1})$, $\theta _1$ is the angle of attack mostly $\theta _{1} = 6^{0}$ or $\theta _{1} = 20^{0}$ then $r \in (0,1)$.

$$\bullet$$ **Case 2** if $$fit(X^{t+1}) \le fit(X_{g}^{t})$$

$\bullet$ **Trailing phase** In the phase where the searcher transitions into a follower role in the Humming Sparrow Optimization model, the individual adapts a strategy of emulating the behavior of the best-performing searcher within the population. This follower behavior is driven by the intention to benefit from the successful exploration patterns and decisions made by the top-performing agents. By tracking and mimicking the movement and choices of the best searcher, the follower seeks to exploit the knowledge and insights that have led to superior solutions. The imitation aims to benefit from successful exploration patterns and decision made by top-performing agents. This adaptive approach allows the follower to leverage the collective intelligence of the swarm, focusing on areas of the search space that have demonstrated success in terms of improved fitness values. The follower phase enhances the overall efficiency of the optimization process by encouraging knowledge transfer and the propagation of effective search strategies throughout the population, ultimately contributing to the convergence of the algorithm toward more optimal solutions.

$$\bullet$$ **condition-1** if $$fit(X^{t+1}) < fit(X_{g}^{t})$$ & $$( R_{2} < ST)$$

$$\bullet$$ **exploitation**

$R_{2}$ alertness probability $\epsilon (0,1)$, *T*- safety threshold values $\epsilon (0.5,1)$

During the follower phase, the follower’s activity is not directly observed or taken into account by the searcher. The follower operates independently, emulating the behavior of the best-performing searcher. The follower operates independently, emulating the behavior of the best-performing searcher. It also involves intensifying search efforts in promising areas of the solution space to further optimize the objective function. Still, the information or decisions the follower makes do not influence or guide the searcher’s actions. This lack of direct interaction between the searcher and the follower preserves the autonomy of each agent in the population. Then,

18 $$\begin{aligned} X^{t+1} = X^{t} e^{\frac{-t}{\alpha -t_{max}}} + \rho X^{t-1} e^{\frac{t}{\alpha (1-\alpha )}}t_{max} \end{aligned}$$

$\alpha \rightarrow \frac{t}{t_{max}}$, $\rho \rightarrow$ frequency of alarm min g signal.

19 $$\begin{aligned} X^{t+1} = x^{t} + rd\lambda \end{aligned}$$

$$rd \rightarrow -1\; to \;1$$, $$\lambda \rightarrow 0.1(\mu -1)$$

Now from (18) and (19)

$$x^{t} + rd\lambda = X^{t} e^{\frac{-t}{\alpha -t_{max}}} + \rho X^{t-1} e^{\frac{t}{\alpha (1-\alpha )}}t_{max}$$

after simplification,

$$X^{t} = \frac{1}{\alpha t} [\rho X^{t-1}(e^{(\frac{-t}{\alpha (1-\alpha )t_{max}})})]$$

where, $\alpha ^{t} = 1-e^{\frac{-t}{\alpha }} t_{max}$ substitute $X^{t}$ in (18)

$$X^{t+1} = \frac{1}{\alpha _{t}} [\rho X^{t-1} (e^{\frac{-t}{\alpha (1-\alpha )t_{max}}})-rd\lambda ] + rd\lambda$$

At instant time $X^{t-1} \equiv X^{t}$

20 $$\begin{aligned} X^{t+1} = \frac{1}{\alpha _{t}} [\rho X^{t} (e^{\frac{-t}{\alpha (1-\alpha )t_{max}}})-rd\lambda ] + rd\lambda \end{aligned}$$

The occurrence of the current position being identical to the previous position in the later stages of convergence signifies a potential state of stagnation in the optimization process. This suggests that the algorithm is reaching a point where further exploration is limited, and the searchers are encountering difficulty in finding new, improved solutions. This convergence may indicate proximity to a local optimum or a region where incremental improvements are challenging. To address this, optimization algorithms often incorporate exploration mechanisms, such as random perturbations, to encourage diversification and prevent premature convergence. The algorithm aims to facilitate continued exploration by introducing randomness, potentially uncovering better solu- tions in the search space. This adaptive strategy helps enhance the algorithm?s ability to escape local optima and converge towards more globally optimal solutions. The effectiveness of optimization algorithm is also enhanced as it prevents premature convergence and promotes discovery of high quality solutions.

$\bullet$ **condition-2** if $fit(X^{t+1}) < fit(X_{g}^{t})$ & $R_{2} \ge ST$ which means the trailing of the follower is found by searcher then

21 $$\begin{aligned} X^{t+1}= & {} X^{t} + \frac{|t_{max - t}|}{|t_{max} + t|} X^{t}(\mu -1)\nonumber \\ X^{t+1}= & {} X^{t}[1 + \frac{|t_{max - t}|}{|t_{max} + t|} (\mu -1)] \end{aligned}$$

Here, $\mu$ and *l* denote the upper and the lower bound.

Also,

22 $$\begin{aligned} X^{t+1} = X^{t} + r_{2}(X_{g}^{t} - MFX^{t}) \end{aligned}$$

Where *MF* is denoted as the mutation factor (1 or 2) $MF = round(1+K(2-1))$, $K = 1$(constant) $X_{g}^{t}$ denotes the global best.

In later stage of convergence,$X_{g}^{t} \equiv X^{t}$

Based on 22 equation, if $MF=1$, then the follower is in the local search phase.

If, $$MF=2$$ then

23 $$\begin{aligned} X^{t+1}= & {} X^{t} -r_{2}X^{t}\nonumber \\ X^{t+1}= & {} X^{t}( -r_{2}) \end{aligned}$$

Equations 21 and 23 likely contain the mathematical rules or conditions that dictate the movement or decision-making process of the follower. The fact that the follower is engaged in a global search suggests that these equations are designed to guide the follower to explore a broader range of solutions, aiming to escape local optima and discover more globally optimal solutions. This behavior is crucial for the effectiveness of optimization algorithms, as it helps prevent premature convergence and promotes the discovery of high-quality solutions in the search space.

24 $$\begin{aligned} X^{t+1}= & {} \frac{ 1}{2} [X^{t+1} + X^{t}]\nonumber \\ X^{t+1}= & {} \frac{ 1}{2} [X^{t}(1 + \frac{|t_{max - t}|}{|t_{max} + t|} (\mu -l) + X^{t}(1-r_{2})]\nonumber \\ X^{t+1}= & {} \frac{ 1}{2} [X^{t}(2 -r_{2} + \frac{|t_{max - t}|}{|t_{max} + t|} (\mu -l)] \end{aligned}$$

**Figure 7:**
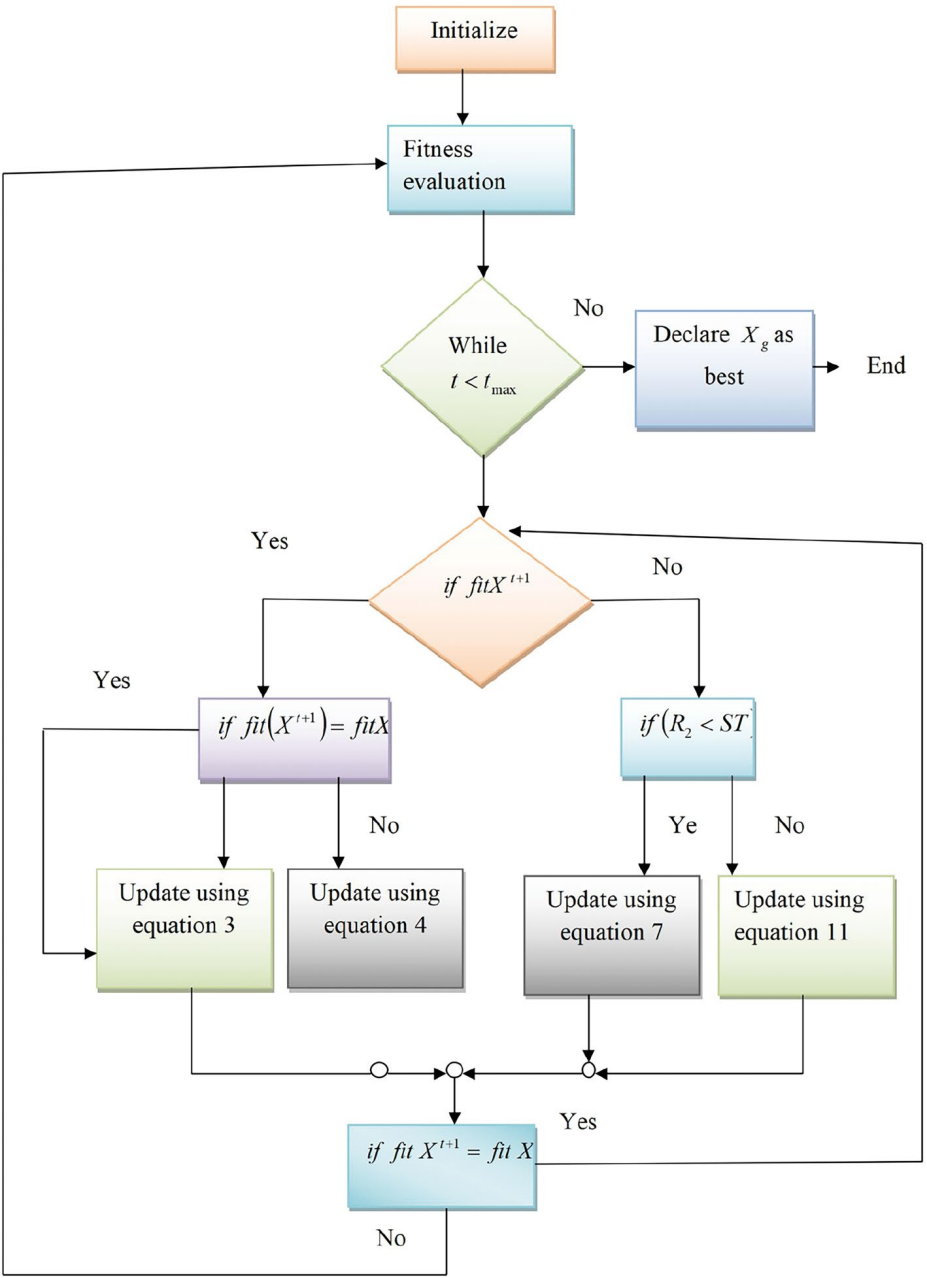
Flowchart for the humming sparrow optimization.

$\bullet$ **Termination** Upon the condition being met, the fitness is reassessed, and the global solution is declared that represents the adaptive tuning parameters of the SADCNN model. Figure 7 shows the Flowchart of the proposed optimization. Optimization enhances the model’s ability to identify subtle correlations in diverse data, ultimately leading to more reliable error corrections. Therefore, the weight and biases of the classifier are tuned by the humming sparrow optimization to enhance the models performance in error correction. Here, the humming sparrow optimization is used to find the best solutions that make the model detect the error efficiently. This optimization tunes the classifiers by modifying the weight and bias with the solution obtained in each iteration and checks the performance of the model. The process keeps going until it finds the best solution and modifies the weight and bias based on the attained best solution to detect the errors more efficiently.

## Result and discussion

The quantum error correction model is developed using the HSO-based SADCNN framework, and its efficacy is compared to alternative approaches.

### Experimental setup

An internal memory capacity of 8GB and the Windows 10 operating system is employed for the experimental setup of the quantum error correction model.

### Performance metrics

#### Accuracy

Quantum error correction accuracy (ACC) is computed as the ratio of the number of correctly corrected quantum states *C* to the total number of quantum states *N* processed:

25 $$\begin{aligned} ACC = \frac{C}{N} \end{aligned}$$

This formula quantifies the precision of the error correction process, with higher accuracy values indicating more effective preservation of quantum information during the correction.

#### Logical error probability

Logical error probability is the probability $(P_{logical})$ that an error affects the logical state of a quantum system even after error correction. It is calculated as the ratio of the number of logical errors $(E_{logical})$ to the total number of corrected states *C*

26 $$\begin{aligned} P_{logical} = \frac{E_{logical}}{C} \end{aligned}$$

A lower logical error probability indicates a more resilient error correction code that better protects the logical qubits from errors.

#### Bit error rate

The Bit Error Rate(BER) in Quantum Error Correction assesses the accuracy of determining classical bits associated with measured quantum states. It is calculated as the ratio of incorrectly determined bits $(B_{err})$ to the total number of bits processed $(B_{total})$:

27 $$\begin{aligned} BER = \frac{B_{err}}{B_{total}} \end{aligned}$$

A lower BER signifies more reliable correction of errors in classical information obtained from quantum measurements, ensuring the fidelity of transmitted classical information.

### Performance analysis concerning TP

**Figure 8:**
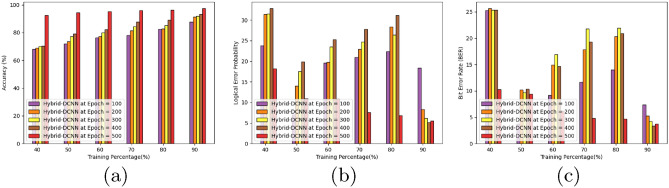
Performance analysis concerning TP (**a**) accuracy, (**b**) logical error rate, and (**c**) bit error.

Figure 8 presents an analysis of the accuracy, logical error probability, and bit error rate for the HSO-based SADCNN models. Utilizing epoch values of 100, 200, 300, 400, and 500 while maintaining a TP of 90%, the HSO-based SADCNN models exhibit increasing accuracy values of 66.59%, 72.23%, 81.74%, 96.64%, and 98.01% (refer to Fig. 8a).

The outcomes from the HSO-based SADCNN models are illustrated in Fig. 8b, revealing minimum logical error probabilities of 18.15, 10.89, 10.81, 7.54, 6.85, and 5.51, achieved at a TP of 90%. Moving to Fig. 8c, which provides a comprehensive summary, the models achieve minimum bit error rates of 10.30, 9.43, 6.20, 4.80, 4.65, and 3.72, also at a TP of 90%. These results underscore the effectiveness of the HSO-based SADCNN models in achieving high accuracy and minimizing both logical and bit error rates under various epoch settings.

### Performance analysis concerning k-fold

**Figure 9:**
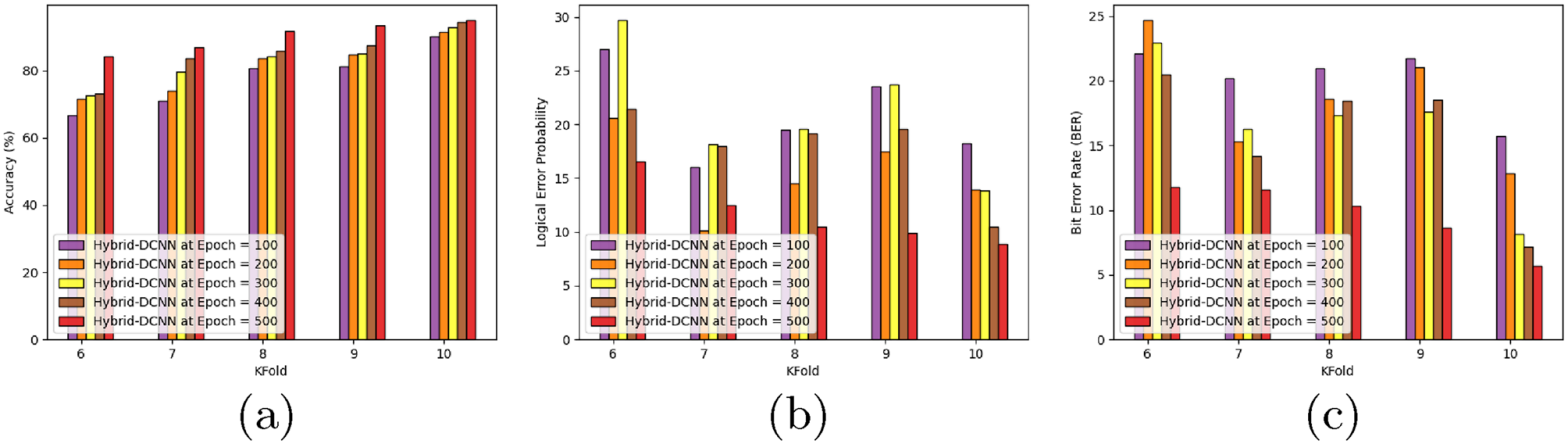
Performance analysis concerning k-fold (**a**) accuracy, (**b**) logical error rate, and (**c**) bit error.

Figure 9 provides a detailed analysis of the accuracy, logical error probability, and bit error rate for the HSO-based SADCNN models. Employing epoch values of 100, 200, 300, 400, and 500 while maintaining a k-fold setting of 10, the HSO-based SADCNN models showcase increasing accuracy values of 90.23%, 91.67%, 92.81%, 94.41%, and 94.99%, as depicted in Fig. 9a.

The outcomes from the HSO-based SADCNN models are presented in Fig. 9b, revealing minimum logical error probabilities of 18.21, 13.92, 13.83, 10.52, and 8.87, achieved during the k-fold 10. Moving to Fig. 9c, which offers a comprehensive summary, the models attain minimum bit error rates of 15.74, 12.85, 8.18, 7.20, and 5.72, all under the k-fold setting of 10. These results highlight the robust performance of the HSO-based SADCNN models across various epochs and k-fold configurations, showcasing high accuracy and effective reduction in logical and bit error rates.

### Comparative methods

A comparative analysis is conducted using a range of models, including logistic regression [25], decision tree [26], ANN [27], deep CNN [28], HBO-DCNN [29] and SSA-DCNN [30]. This comparison aims to illustrate the effectiveness of the HSO-based SADCNN.

#### Comparative analysis concerning TP

**Figure 10:**
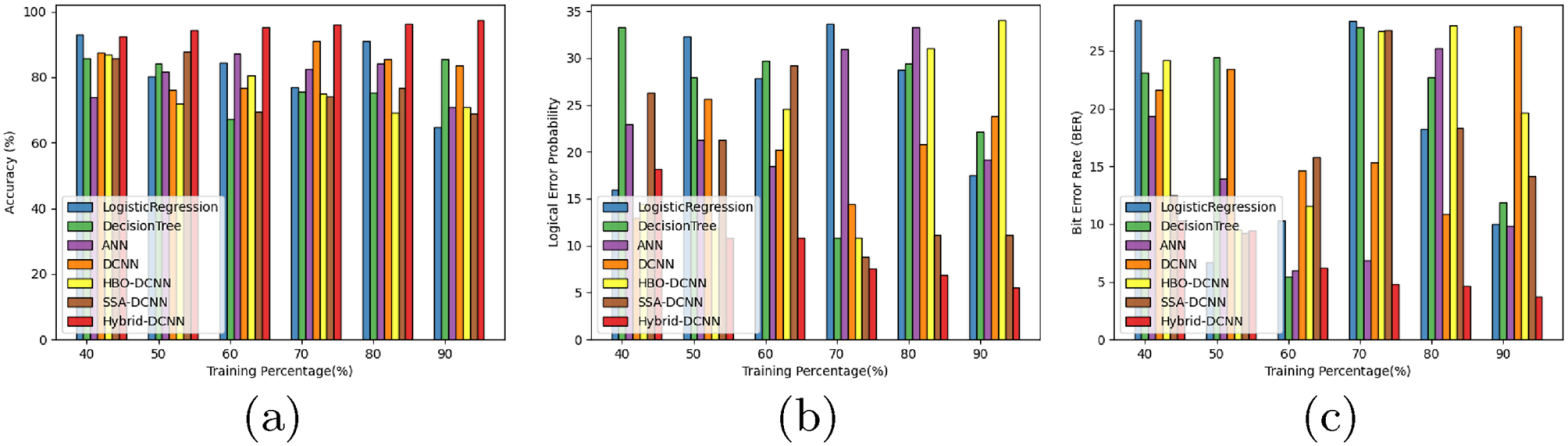
Comparative analysis concerning TP (**a**) accuracy, (**b**) logical error rate, and (**c**) bit error.

The accuracy of the HSO-based SADCNN model for quantum error correction is illustrated in Fig. 10a while ensuring a TP of 90. With a performance that surpasses the SSA-DCNN model by $15.34\%$, the HSO-based SADCNN model achieves an accuracy of $96.31\%$.

Figure 10b displays the logical error probability of the HSO-based SADCNN model in quantum error correction while upholding a TP of 90. Significantly outperforming the SSA-DCNN model by minimum error, the HSO-based SADCNN model demonstrates a logical error probability of 5.51.

Within Fig. 10c, the bit error rate of the HSO-based SADCNN model in quantum error correction is exhibited while upholding a TP of 90. This model outperforms the SSA-DCNN by a margin of minimum error, resulting in the HSO-based SADCNN model achieving a minimum bit error rate of 3.72.

#### Comparative analysis concerning k-fold

**Figure 11:**
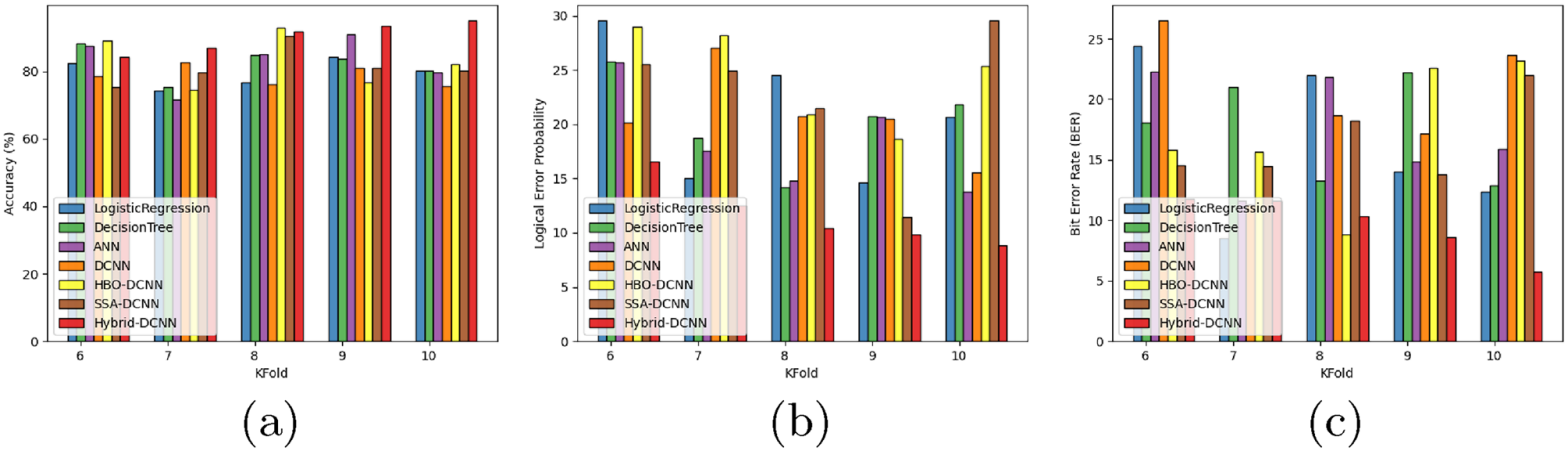
Comparative analysis concerning K-fold (**a**) accuracy, (**b**) logical error rate, and (**c**) bit error.

The accuracy of the HSO-based SADCNN model for quantum error correction is illustrated in Fig. 11a, while ensuring a k-fold of 10. With a performance surpassing the SSA-DCNN model by 15.62% , the HSO-based SADCNN model achieves an accuracy of 94.99% Fig. 11b displays the logical error probability of the HSO-based SADCNN model in quantum error correction while upholding a k-fold 10. Significantly outperforming the SSA-DCNN model by minimum error, the HSO-based SADCNN model demonstrates a logical error probability of 8.87. Within Fig. 11c, the bit error rate of the HSO-based SADCNN model in quantum error correction is exhibited while upholding a k-fold 10. This model outperforms the SSA-DCNN by a margin of minimum error, resulting in the HSO-based SADCNN model achieving a minimum bit error rate of 5.72.

### Analysis based on errors

**Figure 12:**
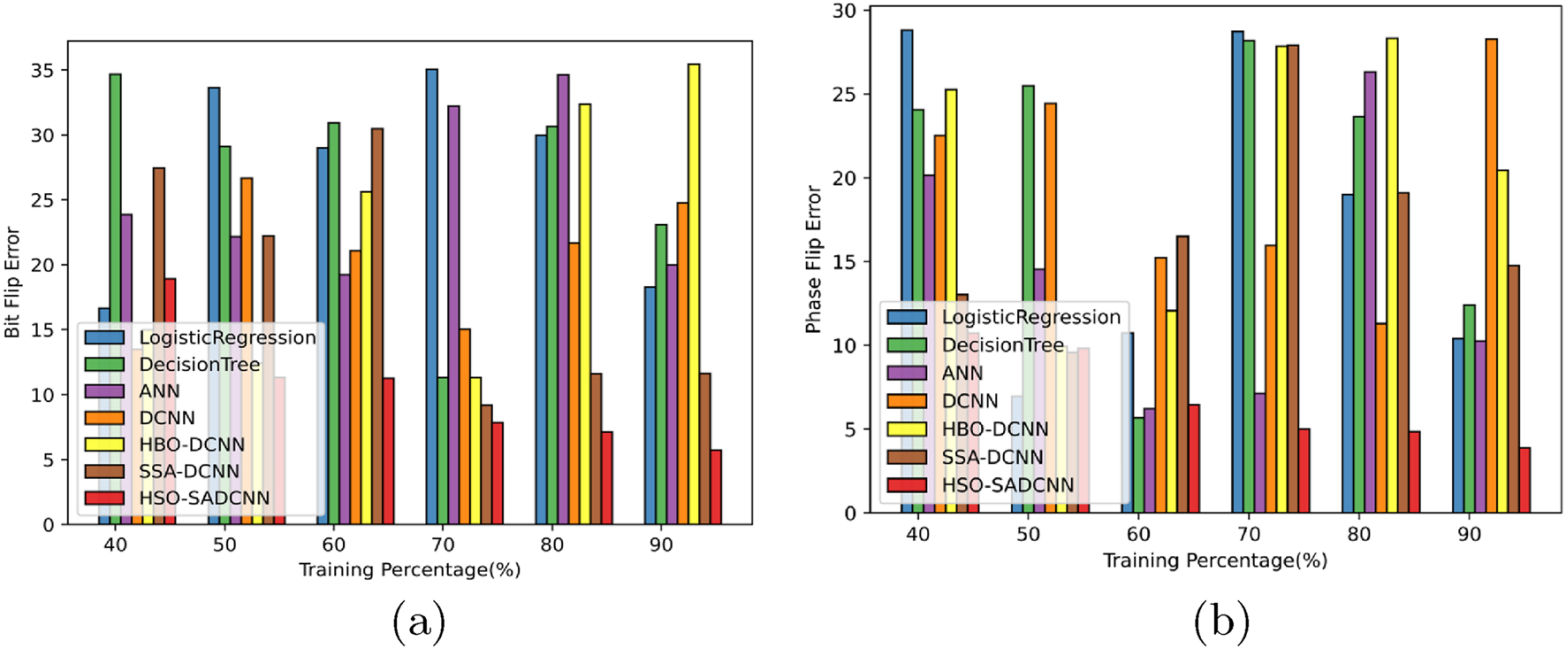
Analysis on error (**a**) bit flip error, (**b**) phase flip errors.

The errors such as bit flip error and phase flip errors are analyzed by the proposed HSO-based SADCNN model and the attained error is compared with the existing methods to show the efficiency of the proposed method. The model attains low error value for both bit flip error and phase flip errors, which are very much less than compared methods and is depicted in Fig. 12. A bit flip error is particularly problematic because it occurs in the measurement basis. This means that bit flips directly influence the information. A phase flip error occurs when the phase of a qubit changes, which is similar to a bit flip in another basis. By detecting these two errors efficiently with low error rate, the effectiveness of the proposed is proved.

### Comparative discussion table

The proposed HSO-based SADCNN model for TP 90% and K-fold 10 are showcased in Tables 1 and 2 respectively, stands out in quantum error correction through a comparative analysis with existing methods. Its distinctive qualities, rooted in the synergy between Harmony Search Optimization and SADCNN, promise improved accuracy, reduced logical error probability, and lower bit error rates. The theoretical justification lies in the efficient solution space exploration of the HSO algorithm and the tailored architecture of SADCNN. This understanding highlights the model’s advantages and guides future research, inspiring the development of more sophisticated quantum error correction models that can harness these principles for enhanced performance.

**Table 1:** Comparative discussion table for TP as 90%.

Models	TP 90		
	Accuracy	LEP	Bit error rate
Logistic regression	64.90	17.53	9.99
Decision tree	85.55	22.15	11.91
ANN	70.80	19.18	9.85
DCNN	83.58	23.76	27.14
0HBO-DCNN	70.80	34.02	19.63
SSA-DCNN	68.83	11.18	14.16
Hybrid-DCNN	97.35	5.51	3.72

**Table 2:** Comparative discussion table for K-fold as 10.

Models	K-fold 10		
	Accuracy	LEP	Bit error rate
Logistic regression	80.15	20.67	12.33
Decision tree	80.15	21.86	12.88
ANN	79.64	13.74	15.87
DCNN	75.53	15.51	23.66
HBO-DCNN	82.21	25.36	23.17
SSA-DCNN	80.15	29.52	22.00
HSO-SADCNN	94.99	8.87	5.72

## Conclusion

The research concludes by presenting a dedicated deep learning-based decoder specifically designed for heavy hexagonal codes in quantum error correction. The proposed decoder, featuring a Self-adaptive Deep CNN Noise Correction Module, is systematically evaluated across various code distances using the Humming Sparrow optimization algorithm. The hybrid optimization approach, combining Humming Bird and Sparrow search optimization, showcases promising results in enhancing the error correction capabilities of the decoder. The integration of herding and tracing characteristics orchestrates a dynamic exploration strategy. The research emphasizes the significance of these findings in addressing the challenges associated with quantum error correction in superconducting qubits. The proposed methodology demonstrates potential improvements in the reliability and accuracy of heavy hexagonal quantum codes for practical quantum computing applications. Based upon the achievements, the metrics for TP 90 demonstrate significant progress, boasting a commendable accuracy rate of $97.35\%$, coupled with reduced logical error probability and a diminished bit error rate, marked at 5.51 and 3.72, respectively. Future work involves integrating quantum reinforcement learning into the decoding process, enabling the SADCNN decoder to adapt dynamically in real-time to evolving quantum error patterns, thereby enhancing its resilience and efficiency in quantum error correction.

### Supplementary Information

Supplementary Information.

## Data Availability

Data are available on request to the corresponding author Ravikumar Bandaru.
