# Supplementary figures and images for: Quantum error-correction using humming sparrow optimization based self-adaptive deep cnn noise correction module
Source: Sci Rep. 2024 Jun 21;14:14289. doi: 10.1038/s41598-024-65182-2 (PMC11192916; doi:10.1038/s41598-024-65182-2)

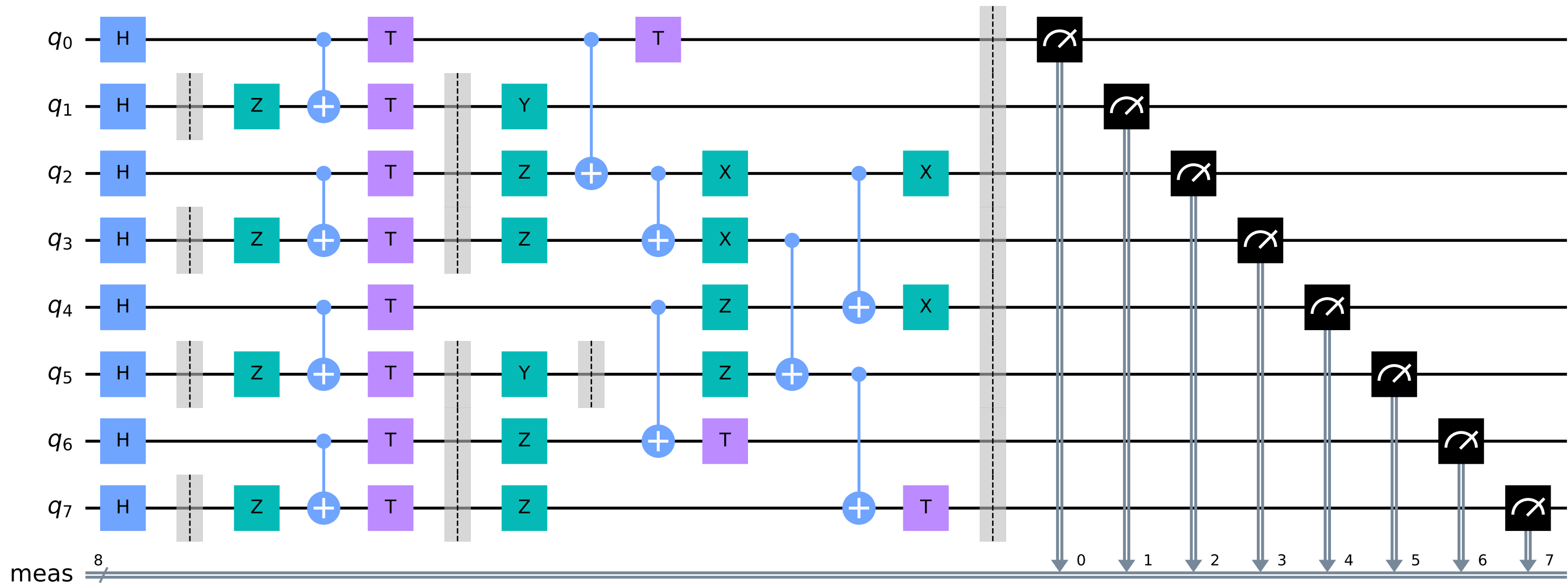

Supplement: Supplementary file 1 — Supplementary Information. [file 41598_2024_65182_MOESM1_ESM.zip › DATA SET.pdf]
